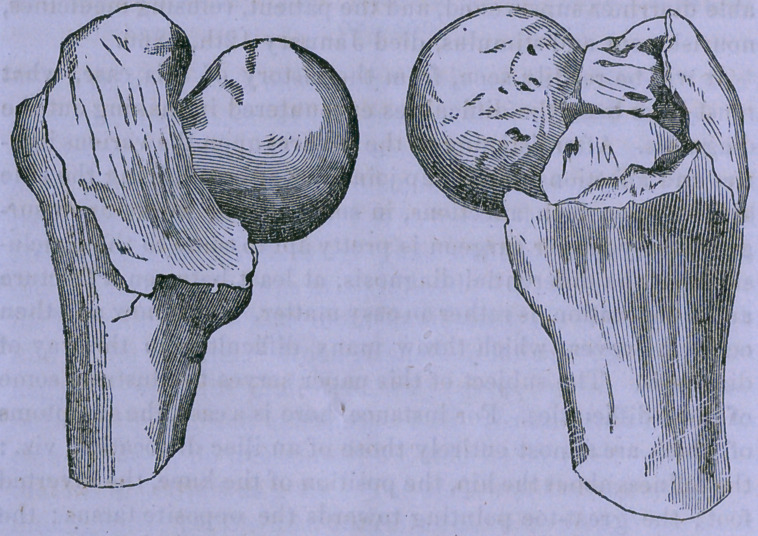# Impacted Fracture of the Neck of the Femur, Etc.

**Published:** 1868-09-01

**Authors:** John E. Owens

**Affiliations:** Surgeon to St. Luke’s Hospital, Chicago


### IMPACTED FRACTURE OF THE NECK OF THE FEMUR SIMULATING ILIAC DISLOCATION.

BY JOHN E. OWENS, M.D., SURGEON TO ST. LUKE'S HOSPITAL, CHICAGO.

W. B., aged 73, a stone-cutter, was admitted to St. Luke's Hospital, December 24th, 1865. About a week before his admission, the patient, upon entering his room, and, in the dark, groping about for the matches, fell over a chair. Some pain was felt in the hip-joint at the time, but almost immediately he got up, struck a light, and went to bed. The patient did not feel that he was at all injured; but, in consequence of his becoming more and more debilitated, his friends brought him to the hospital. Upon admission he was pale, thin and haggard, appetite almost entirely gone, pulse feeble and rather frequent, and extremities cold. He said that he had no pain—indeed, no uneasiness, but that he only felt weak, and that his appetite was gone. The history of the case was given intelligently, yet his own statement gave no clue to the existence of any affection other than debility. The nurse, however, informed me that his thigh was bruised. The leg was found in the following condition: there was considerable ecchymosis about the groin, and extending over two-thirds of the thigh of the affected side; a comparative fullness of the region of the ant. sup. spine, and the trochanter major; the knee a little above, and somewhat in advance of the sound one; the foot inverted; the great toe directed towards the opposite tarsus; the thigh slightly bent upon the pelvis, and the leg upon the thigh; the limb from one-half to two inches shorter than the sound one. Neither whilst recumbent, nor whilst we held him in an upright position, was crepitation elicited; the limb could be moved pretty freely in all directions, yet movement caused considerable pain about the joint; the arc described by the great trochanter, upon rotating the affected limb, seemed about equal to that of the other side; extension applied at the knee did not lengthen the limb; the foot was able to be everted, but, when left to itself, returned to the position already described. We diagnosed impacted fracture of the neck of the femur. The leg was placed in a comfortable position upon pillows. In a few days an intractable diarrhœa supervened, and the patient, refusing medicines, nourishment and stimulus, died January 12th, 1866.

It will be readily seen, from the history of this case, what must have been the difficulties encountered in making out the diagnosis. After listening to the lectures upon the various injuries and affections of the hip-joints, or after studying the able articles upon such affections, in some of our works upon surgery, the younger surgeon is pretty apt to come to the conclusion that the differential diagnosis, at least between a fracture and a dislocation, is rather an easy matter. Cases now and then occur, however, which throw many difficulties in the way of diagnosis. The subject of this paper serves to illustrate some of these difficulties. For instance, here is a case, the symptoms of which are almost entirely those of an iliac dislocation, viz.: the fullness about the hip, the position of the knee, the inverted foot; the great-toe pointing towards the opposite tarsus; the want of crepitation, and the non-restoration of the proper length of the limb by extension.

In making up the diagnosis, four facts are taken into consideration: the patient was an old man, the head of the bone was not felt on the dorsal surface of the ilium, as it can be in those subjects in iliac dislocation; the limb could be moved tolerably freely in all directions, and there was a want of crepitation. In old men—men over 65—owing to its comparative brittleness, the neck of the femur will commonly give way before a dislocation is produced; and, indeed, the forces that, in middle life, usually produce dislocations, in old age produce fractures. In an iliac dislocation, the foot, as mentioned above, is firmly fixed in a constrained manner; but there is no reason, except from the consensual avoidance of pain, why this should be the reason in an impacted fracture. Indeed, in this case, the free motion of the afflicted limb, and the want of crepitation, owing to the firm manner in which the fragments are locked together, were the diagnostic symptoms—and that, too, in spite of the knee and foot assuming a position directly the reverse of that usually attributed to this species of fracture.

We were not able to have an autopsy in the hospital, but one night whilst at work in the dissecting room of one of the colleges, this old man's body was brought in by the resurrectionist. The bone was found in the condition in which the figure represents it, viz.: there are six pieces; the fracture extends through the trochanters; the neck is firmly impacted amongst the surrounding fragments; the upper end of the shaft and one of the larger pieces are somewhat excavated, forming a socket for the broken end of the neck; there was no evidence that the reparative process had commenced amongst the fragments; the pieces were held in excellent position, and any successful efforts to have restored the limb to its normal length and position, would have placed the parts in a position less capable of bony union, than the one in which the autopsy revealed them. It is well known that bony union has taken place in this variety of fracture, and that, too, in patients older than this one. Occurring usually in old and feeble persons, it is better not to disturb the relative position of the impacted fragments; for a shortened, but firm limb is much better than a fracture. In the examination of these cases, the movements of the leg must be conducted with gentleness, lest the neck be displaced from its impacted position. Extension, especially, must be used very cautiously, and rather with a view to discover if the limb is extensible, than to efface any shortening that may exist. Placing the patient upon a firm and comfortable mattress, and the limb on a double-inclined plane (one of pillows is a good one), and the administration of tonics and the most concentrated nourishment, will give us the best results.

## Figures and Tables

**Figure f1:**